# Fecal carriage of extended-spectrum β-lactamase- and carbapenemase-producing Enterobacteriaceae in Egyptian patients with community-onset gastrointestinal complaints: a hospital -based cross-sectional study

**DOI:** 10.1186/s13756-017-0219-7

**Published:** 2017-06-13

**Authors:** H.M. Abdallah, N. Alnaiemi, E.A. Reuland, B.B. Wintermans, A. Koek, A.M. Abdelwahab, A. Samy, K.W. Abdelsalam, C.M.J.E. Vandenbroucke-Grauls

**Affiliations:** 1 Medical Microbiology and Infection Control, VU University Medical Center, Amsterdam, The Netherlands; 2 Laboratory for Medical Microbiology and Public Health, Hengelo, The Netherlands; 3 Department of Microbiology, Faculty of Veterinary Medicine, Zagazig University, Zagazig, Egypt; 4 National Laboratory for Veterinary Quality Control on Poultry Production, Animal Health Research Institute, Dokki, Giza, Egypt; 5 Department of Virology, Faculty of Veterinary Medicine, Zagazig University, Zagazig, Egypt

**Keywords:** Carbapenemase-ESBL-Egypt-resistance-Enterobacteriaceae

## Abstract

**Objectives:**

The aim of this study was to determine the prevalence of extended-spectrum β-lactamase (ESBL) and carbapenemase production among Enterobacteriaceae isolated from ambulatory patients with gastrointestinal complaints admitted to El-Ahrar General Hospital, Zagazig, Egypt in the period between January 2013 and May 2013.

**Methods:**

One hundred and thirteen Enterobacteriaceae isolates were recovered from 100 consecutive Egyptian patients with community–onset gastrointestinal complaints. The fecal samples were plated directly on selective EbSA-ESBL Screening Agar and on MacConkey agar. Isolate identification was performed with matrix-assisted laser desorption ionization time-of-flight mass spectrometry (MALDI-TOF-MS). Screening for ESBLs and carbapenemases production was done by both the automated VITEK®2 system with AST N198 and by disk diffusion method. Real-time PCR and sequencing were used to characterize the resistance genes. Phylogroups of the *E. coli* isolates were determined by a triplex PCR-based method.

**Results:**

Of 100 patients screened for fecal colonization with extended-spectrum β-lactamase -producing Enterobacteriaceae (ESBL-E) and carbapenemase- producing Enterobacteriaceae (CPE), 68 were colonized with ESBL-E whereas five patients were positive for CPE. One hundred and thirteen Enterobacterceae isolates were recovered from 100 fecal samples, they belonged to *E. coli* (*n* = 72), *Klebsiella pneumoniae* (*n* = 23), *Enterobacter cloacae*(*n* = 3), *Salmonella* spp. (*n* = 1) and other Enterobacterceae isolates (*n* = 14). The *bla*_CTX-M_ gene was detected in 89.04% (65/73) of the ESBL-producing Enterobacteriaceae, whereas *bla*_SHV_ and *bla*_TEM_ were detected in 30.14% (22/73) and 19.18% (14/73) respectively. Three out of 5 carbapenem-resistant isolates harbored New Delhi metallo-beta-lactamase (NDM) and 2 produced Verona integron-encoded metallo- beta -lactamase (VIM). Twenty-two (47.83%) of the ESBL positive isolates were multidrug resistant (MDR). Phylogenetic analysis showed that, of the 51 ESBL-EC isolates, 17 belonged to group B2, 13 to group D, 11 to group A and 10 to group B1.

**Conclusions:**

Nearly two-thirds of the Enterobacteriaceae isolates recovered from feces of ambulatory patients with community–onset gastrointestinal complaints admitted to El-Ahrar General Hospital, Zagazig, Egypt were ESBL producers and one in every 20 patients included in our study was colonized by carbapenemase-producing Enterobacteriaceae. These high colonization rates are worrying, therefore prudent antimicrobial use should be adopted in Egyptian community settings.

## Background

Infections due to extended-spectrum beta-lactamase–and carbapenemase-producing Enterobacteriaceae represent a major global health threat [1] since such bacteria are usually resistant to multiple antimicrobial agents and carbapenems are expensive and not always available, especially in less wealthy countries. Infections with these resistant strains are associated with treatment failure, high mortality, and increased healthcare costs [2–4].

ESBL-producing Enterobacteriaceae (ESBL-E) and carbapenemase-producing Enterobacteriaceae (CPE) are incriminated in both nosocomial and community-acquired infection [5, 6]. The fecal carriage rate of ESBL-E has mainly been investigated during nosocomial outbreaks, whilst few studies were conducted in community settings [5, 7]. High community carriage rates were reported in Thailand (69.3%) and China (50.5%) [8, 9]. Lower rates were demonstrated in most European countries (not more than 12%) and North America (less than 2%) [10–13]. In Egypt, data on the prevalence of ESBL-E in the community remain scarce. A study performed in Cairo (urban population) reported high fecal carriage rate (63.3%) of ESBL-E among healthy individuals [14]. To the best of our knowledge, no previous research has been carried out to determine the fecal carriage rate of CPE in the community. In this study, we aimed to assess the prevalence of ESBL-E and CPE among ambulatory patients with community–onset gastrointestinal complaints admitted to El-Ahrar General Hospital, Zagazig (semi-urban population, 65 km from Cairo), Egypt.

## Methods

### Bacterial isolates

This study was performed in the period between January 2013 and May 2013, at El-Ahrar General Hospital, Zagazig, Egypt, a 608-bed hospital affiliated to the Egyptian health ministry. Approximately 1.5 g of feces was collected aseptically from 100 consecutive patients admitted to the hospital with community–onset gastrointestinal complaints (one sample per patient). Fecal samples were obtained for routine diagnosis of the gastrointestinal complaints and in addition to routine testing for gastrointestinal pathogens. The patients had no past history of travel to the Indian subcontinent or to South-Eastern Asia, nor relatives that had recently traveled to those regions. Fecal samples were suspended in 5 mL of saline (0.9%) and 100 ul of each sample was plated directly on selective EbSA-ESBL Screening Agar [15] for the isolation of bacteria resistant to broad-spectrum cephalosporins and on MacConkey agar for the characterization of the dominant isolates. At least one and up to five colonies per agar plate were investigated. Bacteria were identified by the automated Vitek® MS system (BioMérieux, Marcy l’Étoile, France).

### Phenotypic screening and confirmation of ESBL-E and CPE

Antibiotic susceptibility testing was performed by both the automated VITEK®2 system with AST card N198 (BioMérieux, Marcy l’Étoile, France) and by disk diffusion on Mueller-Hinton agar using ceftazidime (30 μg), cefotaxime (30 μg), meropenem (10 μg), and imipenem (10 μg) disks. The AST card antimicrobial agents panel were ampicillin, amoxicillin-clavulanic acid, piperacillin, piperacillin-tazobactam, cefuroxime, cefoxitin, cefepime, ceftazidime, cefotaxime, meropenem, imipenem, ciprofloxacin, norfloxacin, gentamicin, tobramycin, nitrofurantoin, and trimethoprim-sulfamethoxazole. The results of antibiotic susceptibility tests were interpreted according to the clinical breakpoints recommended by Clinical and Laboratory Standards Institute (CLSI) and the Dutch Society of Medical Microbiology [16, 17]. ESBL production was confirmed with the ESBL combination disks (Rosco, Taastrup, Denmark) according to the guidelines of the Dutch Society of Medical Microbiology [17].

Carbapenemases production was confirmed by carbapenemases double disk synergy test [18]. Enhancement of the inhibition zone in the area between the inhibitor-containing disk (boronic acid and/or dipicolinic acid) and any one of the two carbapenems discs used (meropenem and /or imipenem) was regarded as a positive result [17, 19].

### Characterization of β-lactamase-encoding genes

ESBL phenotypes were analyzed for the presence of genes encoding *bla*_TEM_, *bla*_SHV_ and *bla*_CTX-M_ by real-time PCR using primers described before [20–22]. Carbapenem-resistant isolates were tested for the presence of genes encoding *bla*_KPC_, *bla*_NDM_, *bla*_OXA-48_, *bla*_IMP_, and *bla*_VIM_ by multiplex PCRs as previously described [23]. DNA extraction was done by a boiling lysis method as described [24]. The PCR Amplification conditions were described elsewhere [25, 26].

### DNA sequencing analysis

The Purified PCR products of ESBL producers were sequenced with the Sanger ABI 3730 XL automated DNA sequencer by a commercial company (BaseClear, Leiden, The Netherlands). Nucleotide sequences were aligned by the Codon Code Aligner software (Version 5.0.2) and compared to sequences available at the National Center for Biotechnology Information website (www.ncbi.nlm.nih.gov).

### *E-coli* Phylogroups

*E. coli* isolates were segregated into phylogroups (A, B1, B2 or D) by a triplex PCR targeting *chuA, yjaA* and the *TspE4.C2* DNA fragment, as developed by Clermont et al. [27].

### Statistical analysis

All statistical analyses were performed according to Newcombe, Robert G [28].

## Results

Sixty-eight out of 100 feces samples yielded ESBL-E while CPE was identified in 5% of theses samples. Some samples demonstrated the growth of more than one species of Enterobacteriaceae, resulting in 113 isolates available for analysis. ESBLs were identified in 64.61% (73/113) (with 95% confidence interval (CI): 55.44–72.80) of the isolates and five (4.42%) (95% CI: 1.9–9.94) isolates showed carbapenemase activity. The prevalence of different ESBL types among the different Enterobacteriaceae species is shown in Table 1. It was highest in *Klebsiella pneumoniae.* Because only one *Salmonella* strain isolated, which produced CTX-M, we could not estimate the prevalence of ESBL-production in this species. All five carbapenem-resistant Enterobacteriaceae isolates were *K. pneumoniae.*CTX*-*M enzymes were detected in 89.04% (65/73) of the ESBL-producing Enterobacteriaceae, whereas SHV and TEM were detected in 30.14% (22/73) and 19.18% (14/73) respectively. The simultaneous presence of CTX-M, SHV, and TEM was identified in 9 isolates, 12 isolates coproduced CTX-M and SHV, 4 harbored CTX-M and TEM genes, one expressed SHV and TEM genes and 40 produced CTX-M genes only. Seven *E.coli* isolate expressed ESBL phenotype but no TEM, SHV or CTX-M were detected by PCR.

**Table 1:** Prevalence of the different types of ß-lactamase-encoding genes among different Enterobacteriaceae species

Species	No. of isolates	No. of ESBL positive isolates (%)	CTX-M alone	CTX-M + TEM	CTX-M + SHV	TEM + SHV	CTX-M + TEM + SHV
*E.coli*	72	51 (70.83)	38	3	2	0	1
*Klebsiella pneumoniae*	23	19 (82.61)	0	0	10	1	8
*Enterobacter* spp	3	2 (66.66)	1	1	0	0	0
*Salmonella* spp	1	1 (100)	1	0	0	0	0
Other species	14	0(0)	0	0	0	0	0
Total	113	73 (64.61)	40	4	12	1	9

A summary of the distribution of different ß-lactamase-encoding genes among different Enterobacteriaceae is shown in Table 1.

Three out of 5 carbapenem-resistant *K. pneumoniae* harbored *blaNDM* and 2 produced *blaVIM*. No isolate expressed a combination of carbapenemase resistance genes.Of the 65 CTX-M – producing Enterobacteriaceae isolates, 52 (80%) produced CTX-M-15, five (7.7%) produced CTX-M-3, four (6.2%) produced CTX-M-14, one produced CTX-M-27, one produced CTX-M-32, and two, which belonged to CTX-M group 9, remained unidentified.

Antimicrobial susceptibility testing revealed that of the 73 ESBL-positive isolates, 58 (79.45%) expressed co-resistance to trimethoprim/sulfamethoxazole, 48 (65.75%) to quinolones (ciprofloxacin and /or norfloxacin), 33 (45.21%) to aminoglycosides (gentamicin and/or tobramycin) and only one to nitrofurantoin. Twenty-two (30.14%) of the ESBL-positive isolates were multidrug resistant (MDR) (i.e. resistant to at least one agent in three or more classes of antimicrobials (aminoglycosides, quinolones and cotrimoxazole) [29].The antimicrobial resistance pattern of CPE is provided in Table 2

**Table 2:** The antimicrobial resistance pattern of CPE

Sample No.	Gentamicin	Tobramycin	Ciprofloxacin	Norfloxacin	Nitrofurantoin	Cotrimoxazole
1	R^a^	R	S	S	S	R
2	R	R	S	S	S	R
3	R	R	I	R	S	R
4	S^a^	R	R	R	R	R
5	R	R	R	R	S	R

^*a*^ *R* resistant*, S* sensitive

Phylogenetic analysis of *E.coli* isolate revealed that, ESBL-positive isolates were evenly distributed over the different phylogroups, while phylogroup A was underrepresented among ESBL-negative *E. coli* (Table 3)*.*

**Table 3:** Phylogenetic groups of *E-coli* isolates

Phylogroups	No. of ESBL positive *E.coli*	No. of ESBL-negative *E.coli*
A	11	2
B1	10	8
B2	17	5
D	13	6
Total	51	21

## Discussion

This study was conducted to determine the prevalence of extended-spectrum β-lactamase and carbapenemase production among Enterobacteriaceae isolates recovered from the feces of ambulatory patients with community–onset gastrointestinal complaints admitted to El-Ahrar General Hospital, Zagazig, Egypt.

Our findings showed that almost two in every three Egyptian patients with community–onset gastrointestinal complaints are carriers of ESBL-E. Furthermore, nearly 5% of these patients were colonized with CPE. This high rate of colonization with ESBL-E and CPE is most likely due to imprudent antimicrobial use, as in Egypt and other developing countries, antimicrobial agents are readily available and can be purchased as a commodity. What is more, many Egyptian patients receive antimicrobial treatment without the advice or prescription of a physician or other trained health care provider. Also, even when the antimicrobial treatment is officially prescribed, it is almost entirely empirical and not based on individual susceptibility data nor on surveillance data [30].

Overall, the rate of fecal ESBL-E demonstrated in our study is higher than reported from many other countries in Africa, Asia, Europe and North America, but lower than what has been described for Thailand [9–13, 31–34].

The prevalence of fecal ESBL-E found in a study conducted in Cairo by Abdul Rahman and El-Sherif in 2011, was similar to what we observed [14]. In that study, ESBL-E were detected phenotypically, without genotypic confirmation and the prevalence of CPE was not determined.

CTX-M enzymes were the most common ß-lactamases among the Enterobacteriaceae isolates, followed by SHV and TEM. These results are consistent with those of previous studies from other countries around the world [11–13, 35, 36] that reported the dominance of CTX-M over other types of ESBLs. Which CTX-M alleles are dominant may differ by geographical region; we found that CTX-M-15 was the most frequently identified genotype among CTX-M–producing Enterobacteriaceae isolates. These findings confirm the reports of the worldwide spread and predominance of CTX-M-15 [37, 38].

Approximately one in every 20 patients included in our study, was colonized with CPE. This high frequency of carriage of CPE has not been described before in any African country and is quite alarming. It raises public health concern about the efficacy of carbapenems, which are the last resort for treatment of multidrug-resistant enterobacterial infections [39].

The *bla*_*NDM*_ gene was identified in three of the five *Klebsiella pneumoniae* isolates which showed a carbapenem–resistant phenotype while the other two isolates expressed a *bla*_*VIM*_ gene.

Our findings of NDM–producing isolates among patients who had no identified epidemiological link with the Indian subcontinent, the main reservoir of these isolates, along with the recent reports of NDM-producing Enterobacteriaceae derived from Egyptian septicemic patients and from retail chicken meat in Zagazig, Egypt [40, 41], support the hypothesis of the presence of autochthonous NDM-producing strains in the Middle East region [42].

The presence of Enterobacteriaceae producing NDM or VIM has been documented recently in several countries in the Middle East and North Africa [41, 43–45]. All reports, however, are case reports, describing the molecular characteristics of a few strains isolated from clinical specimens in hospitalized patients. Our study is the first that documents the prevalence, hence the size of the problem, in community-dwelling persons.

The antibiotic susceptibility testing revealed that four-fifths of ESBL-positive isolates were also resistant to trimethoprim/sulfamethoxazole, nearly two-thirds also to quinolones, and one-third also to aminoglycosides. Approximately half of the isolates were multidrug resistant, which stresses the public health threat by these isolates, which leave few options for treatment. Nitrofurantoin still had good activity against ESBL-E and CPE.

ESBL- *E. coli* isolates mainly belonged to the virulent groups B2/D, whereas ESBL- negative *E. coli* isolates were nearly equally distributed over the commensal groups A/B1 and the virulent groups B2/D. This finding suggests that there may be a relationship between virulence and resistance determinants, in contrast to the previously suggested trade-off between virulence and resistance [46].

## Conclusion

In conclusion, nearly two third of the Enterobacteriaceae isolates recovered from feces of Egyptian patients in Zagazig region with community–onset gastrointestinal complaints were ESBL producers and one in every 20 patients included in our study was colonized with CPE. These high rates call for better surveillance of resistance in Egypt, and for a more prudent antimicrobial use. Regulation of sales of antimicrobial agents, as recently introduced in India would be a large step forward [47].

## Abbreviations

CLSI: Clinical and laboratory standards institute
CPE: Carbapenemase- producing Enterobacteriaceae.
ESBL: Extended-spectrum β-lactamase.
ESBL-E: Extended-spectrum β-lactamase -producing Enterobacteriaceae.
MALDI-TOF-MS: Matrix-assisted laser desorption ionization time-of-flight mass spectrometry.
MDR: Multidrug- resistant.
NDM: New Delhi metallo-beta-lactamase.
VIM: Verona integron-encoded metallo- beta -lactamase.
